# Authors' response to the letter to the editor on “Tendon modification with percutaneous ultrasound-guided tenotomy using TENEX®: A histological and macroscopic analysis of a bovine cadaveric model.”

**DOI:** 10.1016/j.inpm.2025.100626

**Published:** 2025-08-15

**Authors:** Sayed E. Wahezi, Ugur Yener, Suwannika Palee

**Affiliations:** a Department of Physical Medicine & Rehabilitation, Montefiore Medical Center, Bronx, NY, USA; b Department of Rehabilitation Medicine, Faculty of Medicine, Naresuan University, Phitsanulok, Thailand

The authors thank Sussman et al. for their engagement with our recent publication. We appreciate the opportunity to clarify several points raised.

Contrary to the interpretation in the letter, in our ex vivo model, the paratenon of the bovine Achilles tendon was dissected and removed due to its visibly thicker and denser structure compared to the human Achilles tendon. Among the three components of the bovine tendon, the soleus tendon was selected as the model due to its structural similarity to the human Achilles tendon. Histological analysis also confirmed the presence of residual peritendinous tissue consistent with paratenon structure surrounding the soleus tendon. Loosening or partial separation of this tissue was observed following application of the TENEX® device. These findings were not intentional but represent a secondary mechanical effect, which we reported descriptively.

This study was not intended to model tendinopathy or demonstrate clinical efficacy. Rather, it was designed to examine the macroscopic and histologic changes caused by the TENEX® device under varying exposure times in a controlled cadaveric model. This approach provides a reproducible platform to better understand how procedural parameters influence tissue effects and may help guide future research involving pathological tendons or in vivo conditions.

The authors acknowledge that the therapeutic effects of percutaneous tenotomy are generally attributed to an inflammatory healing cascade. However, the literature cited in the letter is limited, and the biological mechanism remains incompletely understood. One is an observational case series of 20 patients who underwent lateral epicondylitis treatment three years post tenotomy. The study did not differentiate spontaneous healing from tenotomy induced repair. The other is a pilot animal study which evaluated ultrasound tenotomy on 12 rabbit achilles tendons in 2016 and has not been validated since then. Clinically, procedures that rely on pro-inflammatory effects often result in post-procedural pain and often demonstrate delayed improvement, whereas TENEX® tends to produce minimal post procedure discomfort and rapid symptom resolution [[1], [2], [3], [4]]. These clinical findings suggest that a non-inflammatory mechanism of action may play a contributory role [[5], [6], [7], [8], [9]].

The authors never suggested that the paratenon was, or should be, completely disrupted. Rather, the partial detachment observed in our study may reflect a mechanical phenomenon that could be relevant in certain clinical contexts. We agree that the paratenon is constitutively active; however, this makes it a major regulator of tendon homeostasis and, thus, a location that deserves further investigation. With respect to the claim that certain tendons (e.g., the rotator cuff or common extensor tendons) lack a paratenon, we believe this point warrants more precise anatomical clarification, as their reference to make this claim was not supported by an anatomical or histological source While these tendons may not possess a classical paratenon as seen in the Achilles, most are surrounded by peritendinous connective tissues, that may serve analogous structural and regulatory functions [7,[10], [11], [12]].

The TENEX® tool used only disrupted a small part of a large tendon and this is how the authors describe the performance of their tenotomies [1]. Our ex-vivo technique was the same performed in previous clinical studies published with long term data [1,2,13]. Ultrasound guidance was not used, as this study was not intended to replicate in vivo procedures but rather to evaluate tissue response in a bench-top model.

Lastly, there is no standard of care for the use of the TENEX® device, and the authors were merely attempting to begin objective discussion around this fascinating technology that deserves more scientific discovery.

## Conflict of interest

All authors declared “no conflict of interest” for this publication.
